# Partial involvement of Nrf2 in skeletal muscle mitohormesis as an adaptive response to mitochondrial uncoupling

**DOI:** 10.1038/s41598-018-20901-4

**Published:** 2018-02-05

**Authors:** Verena Coleman, Piangkwan Sa-Nguanmoo, Jeannette Koenig, Tim J. Schulz, Tilman Grune, Susanne Klaus, Anna P. Kipp, Mario Ost

**Affiliations:** 1 Department of Physiology of Energy Metabolism, German Institute of Human Nutrition Potsdam-Rehbrücke, 14558 Nuthetal, Germany; 2 Department of Molecular Toxicology, German Institute of Human Nutrition Potsdam-Rehbrücke, 14558 Nuthetal, Germany; 3 Department of Adipocyte Development and Nutrition, German Institute of Human Nutrition Potsdam-Rehbrücke, 14558 Nuthetal, Germany; 4 Department of Physiology, Chang Mai University, Chang Mai, Thailand; 5 Department Molecular Nutritional Physiology, Friedrich Schiller University Jena, Jena, 07743 Germany

## Abstract

Mitochondrial dysfunction is usually associated with various metabolic disorders and ageing. However, salutary effects in response to mild mitochondrial perturbations have been reported in multiple organisms, whereas molecular regulators of cell-autonomous stress responses remain elusive. We addressed this question by asking whether the nuclear factor erythroid-derived-like 2 (Nrf2), a transcription factor and master regulator of cellular redox status is involved in adaptive physiological responses including muscle mitohormesis. Using a transgenic mouse model with skeletal muscle-specific mitochondrial uncoupling and oxidative phosphorylation (OXPHOS) inefficiency (UCP1-transgenic, TG) we show that additional genetic ablation of Nrf2 abolishes an adaptive muscle NAD(P)H quinone dehydrogenase 1 (NQO1) and catalase induction. Deficiency of Nrf2 also leads to decreased mitochondrial respiratory performance although muscle functional integrity, fiber-type profile and mitochondrial biogenesis were not significantly altered. Importantly, Nrf2 ablation did not abolish the induction of key genes and proteins of muscle integrated stress response including the serine, one-carbon cycle, and glycine synthesis (SOG) pathway in TG mice while further increasing glutathione peroxidase (GPX) activity linked to increased GPX1 protein levels. Conclusively, our results tune down the functions controlled by Nrf2 in muscle mitohormesis and oxidative stress defense during mitochondrial OXPHOS inefficiency.

## Introduction

Skeletal muscle mitochondrial dysfunction due to impaired oxidative phosphorylation (OXPHOS) is a well-established cause of mitochondrial and metabolic disorders^1,2^ as well as aging^3^. Noteworthy, mitochondrial OXPHOS deficiency is often associated with increased free radical and reactive oxidant species (ROS) production by the mitochondria itself^4^ or indirectly by the endoplasmic reticulum (ER) surface^5^. As ROS-induced damage of biomolecules can have detrimental effects on cellular physiology and homeostasis, oxidative stress is assumed to play a crucial role in mitochondrial diseases^6^ and numerous age-related disorders, respectively^7^. In contrast, caloric restriction and physical exercise mildly induce mitochondrial ROS production, but also extend health and lifespan, a phenomenon termed mitohormesis^8,9^. Thereby, cellular stress resistance is mediated by cell-autonomous stress response mechanisms such as activation of endogenous antioxidant defense system, mitochondrial biogenesis and ER unfolded protein response (UPR) signaling cascades^10–12^.

Organisms have a highly conserved muscle oxidative stress defense system to counteract ROS-induced damage^13,14^. As master regulator of cellular redox homeostasis and intermediary metabolism, the transcription factor nuclear factor erythroid-derived-like 2 (Nrf2) binds to the antioxidant/electrophile response element (ARE/EpRE) of target genes and increases the expression of a variety of antioxidant enzymes and enzymes involved in glutathione (GSH) synthesis^15,16^. Thereby, Nrf2 is required for an adaptive cytoprotective response to oxidative stress^17^. After skeletal muscle injury, Nrf2 activity is required for muscle regeneration and effective healing by modulating satellite cell proliferation^18^ as well as protection from excessive inflammation-induced muscle wasting and fibrosis^19^. Upon aging, disruption of Nrf2-ARE/EpRE signaling markedly increases muscle ROS production, oxidative stress and pro-apoptotic signaling^20^. During exercise, activation of Nrf2 is required for the induction of mitochondrial biogenesis and antioxidant response^21,22^. Moreover, *in vitro* studies on murine neurons and embryonic fibroblasts showed that Nrf2 regulates cellular energy metabolism by channeling energy substrates to mitochondrial respiration^23^. Recent studies further suggested a role for the Nrf2 pathway in maintaining mitochondrial integrity via regulation of selected mitophagy induction^24,25^. However, the regulatory function of muscle Nrf2 in the retrograde and adaptive mitochondrial stress response is unknown.

The UCP1-transgenic mouse is a model for skeletal muscle-specific mitochondrial OXPHOS inefficiency^26^ through ectopic overexpression of uncoupling protein 1 (UCP1)^27^. We and others have previously demonstrated that muscle-targeted respiratory uncoupling promotes healthy aging phenotype^28,29^. Consistent with the mitohormesis concept, this was related to an induced endogenous antioxidative defense system^30,31^ as well as a robust cell-autonomous transcriptional and metabolic remodeling^32^, including components of amino acid starvation response, the serine, one-carbon cycle, and glycine synthesis (SOG) pathway, ER-stress/UPR and integrated stress response (ISR) signaling. A similar muscle-specific mitochondrial stress response was observed in a mouse model (Deletor mouse)^33,34^ for mitochondrial myopathy and in patients with adult-onset of mitochondrial disease^35^. Besides, the SOG pathway is well-known to support numerous metabolic processes that are crucial for growth and survival of cancer cells including nucleotide synthesis, glutathione synthesis and NADPH generation for antioxidant defence^36^. Recently, the metabolic reprogramming of cancer cells, and specifically the induction of the SOG pathway, has been shown to be driven by Nrf2 action^37^. Whether Nrf2 is also required for SOG pathway induction and antioxidant response during muscle mitochondrial stress adaptation remains to be elucidated.

In this study, we directly tested for the relevance of Nrf2 under conditions of chronic muscle mitochondrial OXPHOS deficiency using UCP1-transgenic^26^ (TG) mice as a mouse model of muscle-specific mitohormesis crossbred with Nrf2-knockout^38^ (Nrf2-Ko) mice. We performed comprehensive physiological as well as skeletal muscle phenotyping of young/adult mice with particular focus on muscle function and mitochondrial integrity, the SOG/ISR pathway induction as well as the endogenous antioxidant stress defense system.

## Results

### Generation and phenotypic characterization of TG/Nrf2-Ko animals

**Figure 1:**
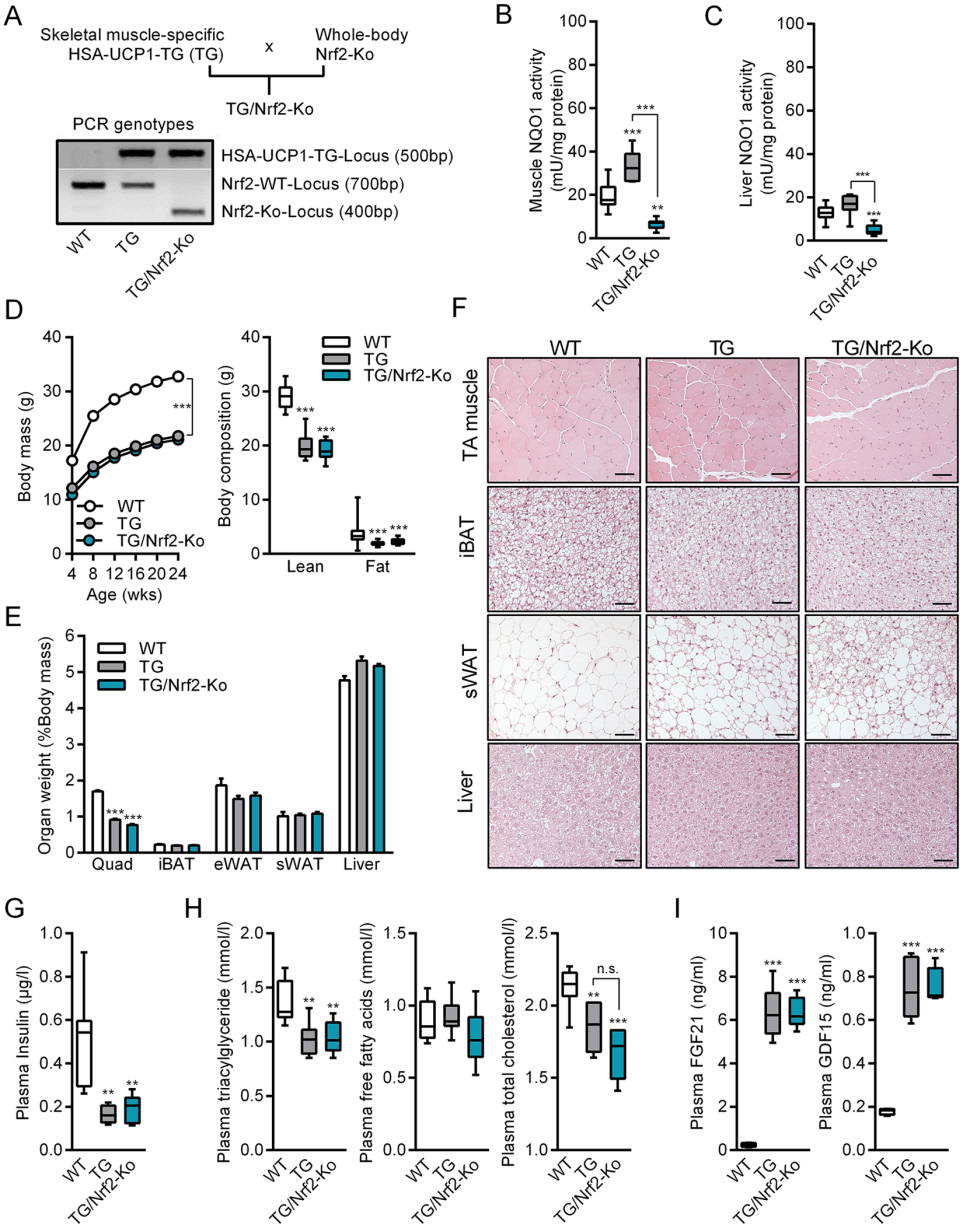
Phenotypic characterization of male TG/Nrf2-Ko animals. (**A**) Scheme of crossbreeding and PCR genotyping of skeletal muscle specific HSA-UCP1-transgenic mice (TG) with whole body Nrf2-Knockout (Nrf2-Ko) mice to generate TG/Nrf2-Ko animals from different gels processed in parallel. (**B**) Quadriceps (Quad) muscle (**C**) liver NAD(P)H quinone dehydrogenase 1 (NQO1) activity as prototypic Nrf2-target and proof of principle for functional ablation of Nrf2 action (n = 7–10). (**D**) Body mass development from 4 to 24 wks and final body composition at 24 wks of age (**E**) Relative Quad, interscapular brown adipose tissue (iBAT), epidydimal (eWAT) and subcutaneous white adipose tissue (sWAT) and liver weights (**F**) Representative H&E histological staining of tibiales anterior (TA) muscle, iBAT, sWAT and liver, bars represent 50 µm. (**G**) Random fed plasma insulin levels and (**H**) plasma triacylglycerides, free fatty acids and total cholesterol (n = 5–9). (**I**) Circulating plasma levels of FGF21 and GDF15 (n = 4–5). All data from 24 wks old male mice. All absolute values are expressed as box-and-whisker plots, relative or normalized data are expressed as means + SEM; **p < 0.01; ***p < 0.001.

We set out to investigate the role of Nrf2 in skeletal muscle mitochondrial stress adaptation. To this end, we generated TG/Nrf2-Ko mice (and WT, Nrf2-Ko, and TG mice) and initially performed a phenotypic characterization of female and male mice fed a standard chow diet up to an age of 12 and 24 weeks, respectively. The genotypes were determined by PCR (Fig. 1A) and further confirmed by enzyme activity analysis of the Nrf2 target NAD(P)H quinone dehydrogenase 1 (NQO1) as a key readout parameter for Nrf2 activity^39^. Notably, mild mitochondrial stress, through respiratory uncoupling, strongly increased NQO1 activity in skeletal muscle of TG mice, but not in liver (Fig. 1B and C). As expected, NQO1 activity was markedly decreased in muscle and liver of both Nrf2-Ko and TG/Nrf2-Ko mice (Figs 1B and C, S1A and S1B). Consistent with previous studies^40,41^, female and male Nrf2-Ko mice, respectively, displayed no obvious phenotypic differences compared to WT mice regarding body mass, body composition, organ weights, plasma insulin, plasma lipids or circulating cytokines at 12 or 24 weeks of age, respectively (Figs S1C–S1H and S2A–S2D). Therefore, we decided to focus on WT and TG mice only as control groups when characterizing the phenotype of TG/Nrf2-Ko mice.

As already described before^28,32^, muscle mitochondrial uncoupling promoted a strong reduction of body mass in young male and female TG mice (Figs 1D and S2A). This was mainly based on a reduced lean body mass, in particular skeletal muscle mass (Figs 1E and S2B, quadriceps muscle). Histological hematoxylin and eosin (H&E) staining confirmed a reduction in muscle fiber size of tibialis anterior (TA) muscle in TG mice^42^ as well as the presence of clusters containing multilocular adipocytes in subcutaneous white adipose tissue (sWAT)^43,44^, a cellular process termed WAT browning^45,46^. Besides muscle and sWAT, morphology of interscapular brown adipose tissue (iBAT) and liver revealed no obvious genotypic differences (Figs 1F and S2D). As reported previously^43^, plasma insulin as well as plasma lipids including triacylglycerides and total cholesterol were significantly reduced, whereas circulating levels of fibroblast growth factor 21 (FGF21) and growth differentiation factor 15 (GDF15), novel biomarkers of mitochondrial stress and disease^32,47–49^, were highly induced in TG mice (Figs 1G–I and S2C). However, TG versus TG/Nrf2-Ko mice were indistinguishable at 12 or 24 wks of age regarding the above described phenotypic characteristics (Figs 1D–I and S2A–D). As there were no obvious gender-specific differences, we only included male WT, TG and TG/Nrf2-Ko mice in further analyses of skeletal muscle.

### Skeletal muscle functional integrity and mitochondrial biogenesis in TG/Nrf2-Ko mice

**Figure 2:**
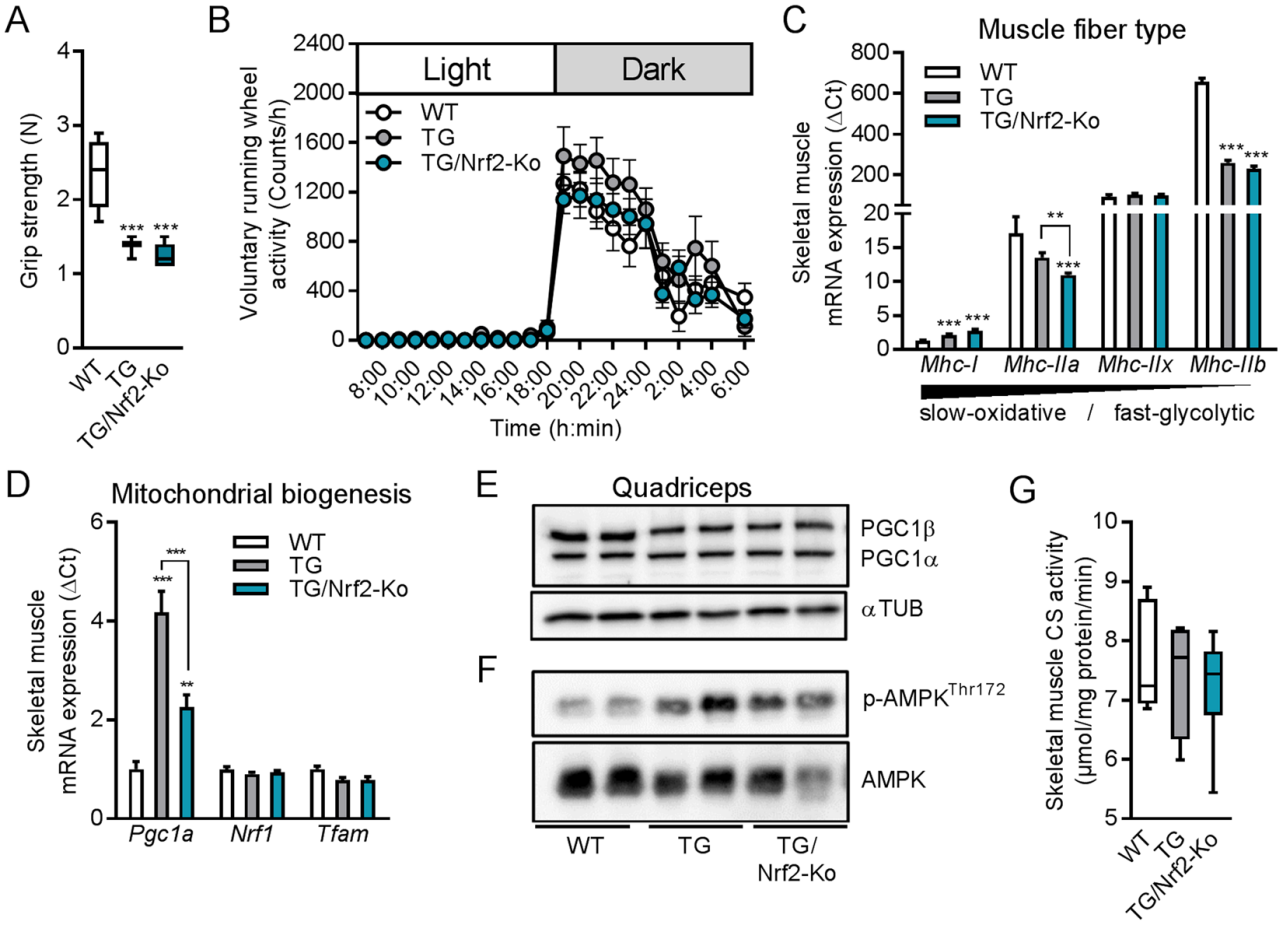
Skeletal muscle integrity and mitochondrial biogenesis in male TG/Nrf2-Ko mice. (**A**) Grip strength of 24 wks old animals (n = 8–10). (**B**) 24 h voluntary running wheel activity of 14 wks old male mice (n = 9–10). (**C**) Skeletal muscle mRNA expression in quadriceps muscle of the myosin heavy chain (*Mhc*) fiber type markers and (**D**) mitochondrial biogenesis markers of 24 wks old animals (n = 8–10). (**E**) Representative immunoblots of PGC1α/β in quadriceps muscle from 24 wks old mice from cropped blots of the same gel using αTubulin (αTUB) as loading control (2 samples shown out of 4–5 analyzed per group). (**F**) Representative immunoblots of phospho-AMPK^Thr172^ and total AMPK in quadriceps muscle from 24 wks old mice from different gels processed in parallel (2 samples shown out of 4–5 analyzed per group). (**G**) Skeletal muscle (gastrocnemius) citrate synthase activity of 24 wks old animals (n = 4–8). All absolute values are expressed as box-and-whisker plots, relative or normalized data are expressed as means + SEM; **p < 0.01; ***p < 0.001.

Recent studies showed that Nrf2 is activated during acute exercise and required for increases in muscle mitochondrial biogenesis gene expression^21^ while this can be compensated during chronic endurance exercise in mice^50^. Here, we assessed the impact of Nrf2 action on skeletal muscle integrity and function following mild muscle mitochondrial stress. Grip strength - as an indicator for skeletal muscle force - was reduced in both TG and TG/Nrf2-Ko mice compared to WT (Fig. 2A). Notably, grip strength of unchallenged Nrf2-Ko mice was not affected (Fig. S1I). Voluntary running wheel activity showed no differences between the genotypes (Fig. 2B). Profiling of muscle fiber type gene expression in quadriceps muscle confirmed a fast-to-slow fiber type switch of TG mice^27^ with increased gene expression of myosin heavy chain I (*Mhc-I*, slow-oxidative) and reduced *Mhc-IIb* (fast-glycolytic). This was preserved in TG/Nrf2-Ko mice (Fig. 2C), although the intermediate isoform *Mhc-IIa* was significantly reduced in those animals compared to WT or TG controls. Mitochondrial biogenesis-associated mRNA expression of peroxisome proliferator-activated receptor gamma coactivator 1-alpha (*Ppargc1a* or *Pgc1α)* was induced in TG mice which was slightly attenuated in TG/Nrf2-Ko mice (Fig. 2D). Moreover, muscle mRNA expression levels of nuclear respiratory factor 1 (*Nrf1*) and mitochondrial transcription factor A (*Tfam*) were neither affected by mitochondrial uncoupling nor Nrf2-deficiency. In contrast to mRNA levels, we were unable to detect any genotype differences in skeletal muscle protein levels of PGC1α/β, whereas AMPK protein phosphorylation was induced in both TG and TG/Nrf2-Ko mice (Fig. 2E and F), confirming our previous results on mitochondrial uncoupling-induced AMPK activation^42^. Additionally, protein levels of mitochondrial respiratory complexes in quadriceps muscle were unchanged between the genotypes (data not shown). Consistent with this, muscle citrate synthase (CS) activity as marker for muscle mitochondrial capacity was not different between the genotypes (Fig. 2G).

### Skeletal muscle mitochondrial respiratory capacity in TG/Nrf2-Ko mice

**Figure 3:**
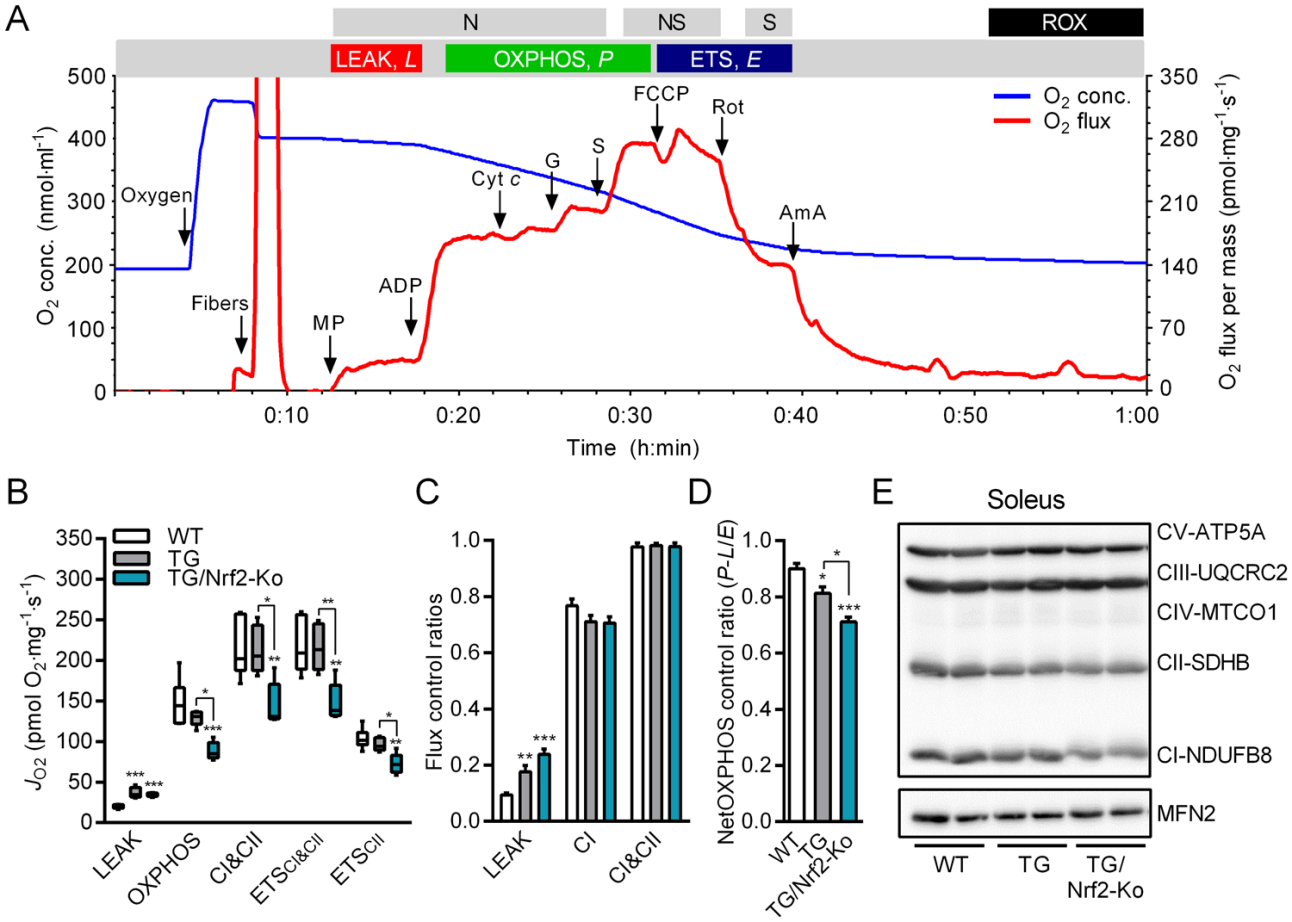
Skeletal muscle fiber mitochondrial respiratory capacity in male TG/Nrf2-Ko mice. (**A**) Representative trace of oxygen (O_2_) concentration (blue line) and O_2_ flux (red line) during substrate-uncoupler-inhibitor-titration (SUIT) protocol for mitochondrial (mt) respiratory capacity in permeabilized mouse soleus muscle fibers, NADH-linked substrates (N-pathway, N), combined with succinate (NS-pathway, NS) and CI-inhibitor rotenone (S-pathway, S): Oxygen (elevation of O_2_ levels in respirometry chambers with permeabilized muscle fibers to avoid the development of an artificial hypoxic or anoxic core in the nonperfused fiber bundle), Fibers (1–2 mg saponin-permeabilized fibers), MP (Malate + Pyruvate; LEAK respiration), ADP (N-OXPHOS capacity), Cyt *c* (cytochrome c, integrity of outer mt-membrane), G (glutamate; N-OXPHOS capacity), S (succinate; NS-OXPHOS capacity), FCCP (NS-ETS capacity, ETS_CI&CII_), Rot (rotenone; S-ETS capacity, ETS_CII_), Ama (antimycin A; less than 2% residual oxygen consumption, ROX). (**B**) Mitochondrial respiratory capacity (*J*_O2_) of oxidative soleus skeletal muscle fibers of 40 wks old male animals (n = 4–5) and (**C**) corresponding flux control ratios and O_2_ fluxes normalized to ETS_CI&CII_. (**D**) NetOXPHOS control ratio (≈(*P − L*)/*E*, expresses the OXPHOS capacity (corrected for LEAK respiration) as a fraction of ETS capacity). (**D**) Representative immunoblots of respiratory chain complexes in soleus muscle of 40 wks old male animals using Mitofusin2 (MFN2) as loading control (2 samples shown out of 4–5 analyzed per group). All absolute values are expressed as box-and-whisker plots, relative or normalized data are expressed as means + SEM; *p < 0.05; **p < 0.01; ***p < 0.001.

Previously, Holmström *et al*. showed that respiration and ATP levels are decreased in neuronal cells and mitochondria isolated from Nrf2-Ko mice^23^. To determine whether mitochondrial respiratory capacity (per tissue wet weight) during mild muscle mitochondrial stress is affected by the genetic ablation of Nrf2, we studied freshly dissected (oxidative) soleus muscle from adult male mice using high-resolution respirometry^51^. Therefore, we prepared saponin-permeabilized muscle fibers and performed a multiple substrate-uncoupler-inhibitor titration (SUIT) protocol^52,53^ for analysis of oxidative phosphorylation (Fig. 3A, representative trace of WT soleus muscle). Non-phosphorylating LEAK respiration (oxygen flux after addition of malate and pyruvate with no ADP present) was increased in chronic respiratory uncoupled soleus muscles of TG and TG/Nrf2-Ko mice confirming the functional transgenic UCP1 overexpression^54^ (Fig. 3B). Following the addition of ADP in saturating concentrations, the OXPHOS state and combined maximum coupled respiration through both complex I and II (CI&CII, after addition of glutamate and succinate) was significantly lower in TG/Nrf2-Ko versus WT or TG mice (Fig. 3B). Finally, maximum electron transfer system capacity (ETS_CI&CII_, following addition of the exogenous uncoupler FCCP) as well as submaximal ETS_CII_ respiratory state (after complex I inhibition by adding rotenone) were also reduced in soleus of TG/Nrf2-Ko mice. The flux control ratio (FCR) at each respiratory state confirmed the higher LEAK respiration and showed a slightly but not significantly lower CI efficiency for both TG and TG/Nrf2-Ko animals while the FCR for CI&CII was unchanged between the genotypes (Fig. 3C). Additionally, netOXPHOS control ratio as a readout of OXPHOS capacity (as fraction of ETS capacity corrected for LEAK respiration) is lowest in TG/Nrf2-Ko versus TG or WT mice (Fig. 3D). Additionally, protein expression of mitochondrial respiratory complex I (CI-NDUFB8) was slightly reduced but not significant in TG/Nrf2-Ko mice (Fig. 3E), whereas protein levels of complex II-IV revealed no differences.

### Ablation of Nrf2 does not prevent the induction of muscle ER-stress and key features of mitohormesis profile

**Figure 4:**
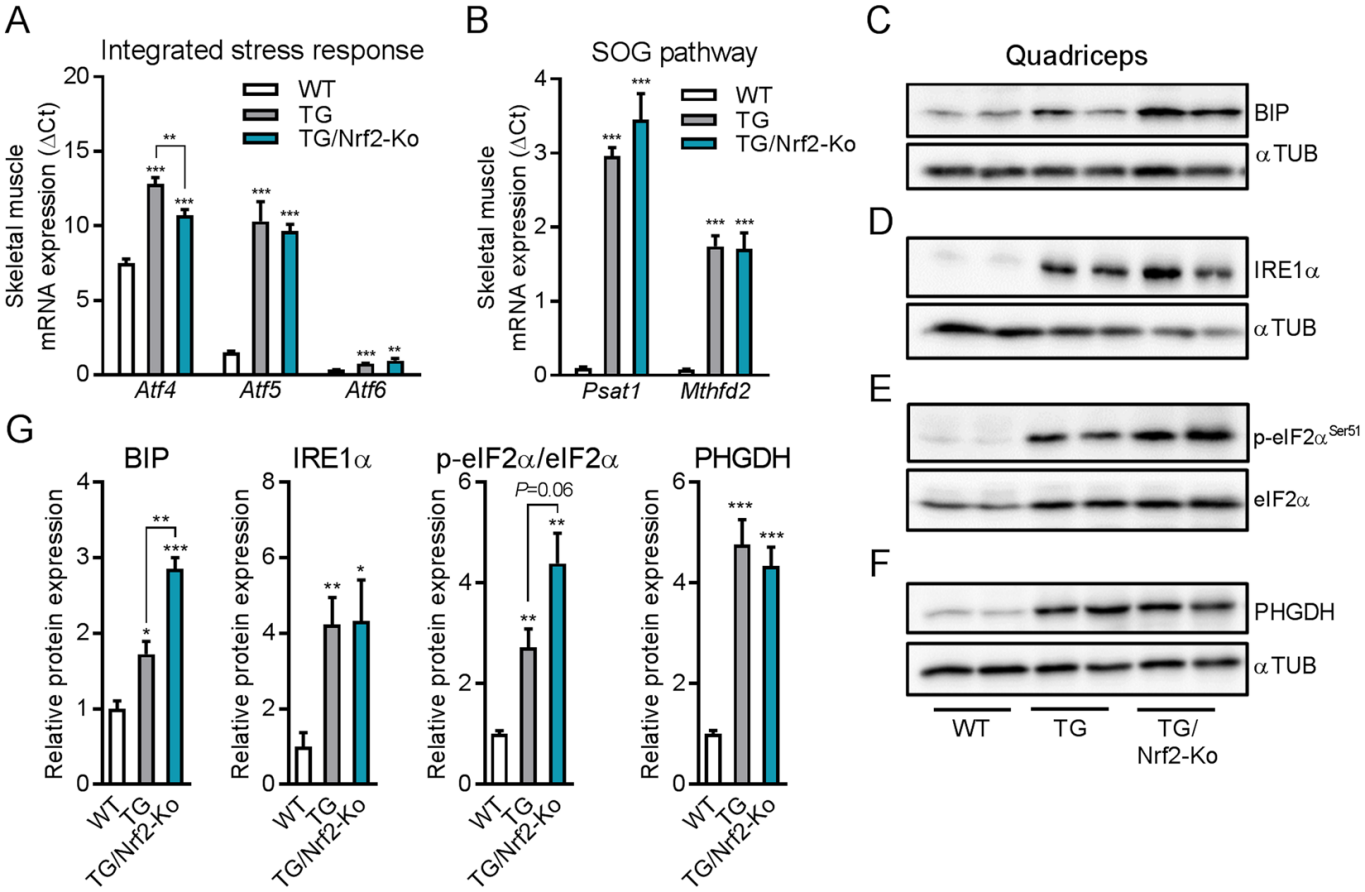
Skeletal muscle expression profile of ER-stress and integrated stress response. (**A**) Skeletal muscle mRNA expression in quadriceps muscle integrated mitochondrial stress response (ISRmt) transcription factors and (**B**) genes of the serine, one-carbon cycle, and glycine synthesis (SOG) pathway (n = 8–10). (**C**–**F**) Representative immunoblots of BIP, IRE1α, and PHGDH from cropped gels using αTubulin (αTUB) as loading control and phospho-eif2α^Ser51^ and total eif2α of one uncropped gel in quadriceps muscle from 24 wks old mice (2 samples shown out of 4–5 analyzed per group). (**G**) Quantification of integrated stress response (ISR) and SOG proteins in quadriceps muscle from 24 wks old mice (4–5 per group). Relative or normalized data are expressed as means + SEM; *p < 0.05; **p < 0.01; ***p < 0.001.

Mitochondrial perturbations trigger various adaptive stress signaling cascades. Recent studies have uncovered a common muscle metabolic remodeling cross-linking integrated stress response (ISR) signaling with serine, one-carbon cycle, and glycine synthesis (SOG) pathway in response to mitochondrial OXPHOS inefficiency^32^ or mitochondrial diseases^34,35,55^. Interestingly, a recent study demonstrated that Nrf2 regulates a serine/glycine biosynthesis metabolic adaptation in cancer cells^37^. Thus, we aimed to evaluate if Nrf2 plays a similar role in skeletal muscle adaptive ISR induction. As shown before^32,44^, we observed a strong induction of mRNA expression of ISR-associated *Atf4, Atf5 and Atf6* in skeletal muscle of the TG mice which was preserved in TG/Nrf2-Ko mice (Fig. 4A). Furthermore, phosphoserine aminotransferase 1 (*Psat1)* and methylenetetrahydrofolate dehydrogenase 2 *(Mthfd2) -* key genes of the SOG pathway - remained highly induced despite the loss Nrf2 action (Fig. 4B). In agreement with the mRNA expression data, protein levels of the critical endoplasmic reticulum (ER) chaperone binding immunoglobulin protein (BIP, also known as 78 kDa glucose-regulated protein, GRP-78) and the unfolded protein response (UPR) sensor inositol-requiring enzyme 1 alpha (IRE1α) were still strongly increased in TG/Nrf2-Ko mice (Fig. 4C,D and G). Furthermore, phosphorylation of eukaryotic initiation factor 2 alpha (eIf2α) as an essential factor for ISR and UPR induction was in tendency even higher in respiratory uncoupled muscle without functional Nrf2 signaling (Fig. 4E and G). Moreover, the induction of phosphoglycerate dehydrogenase (PHGDH), which catalyzes the first and rate-limiting step in serine biosynthesis, was not suppressed in muscle of TG mice by the ablation Nrf2 (Fig. 4F and G).

### Involvement of Nrf2 in antioxidant response during muscle mitohormesis

**Figure 5:**
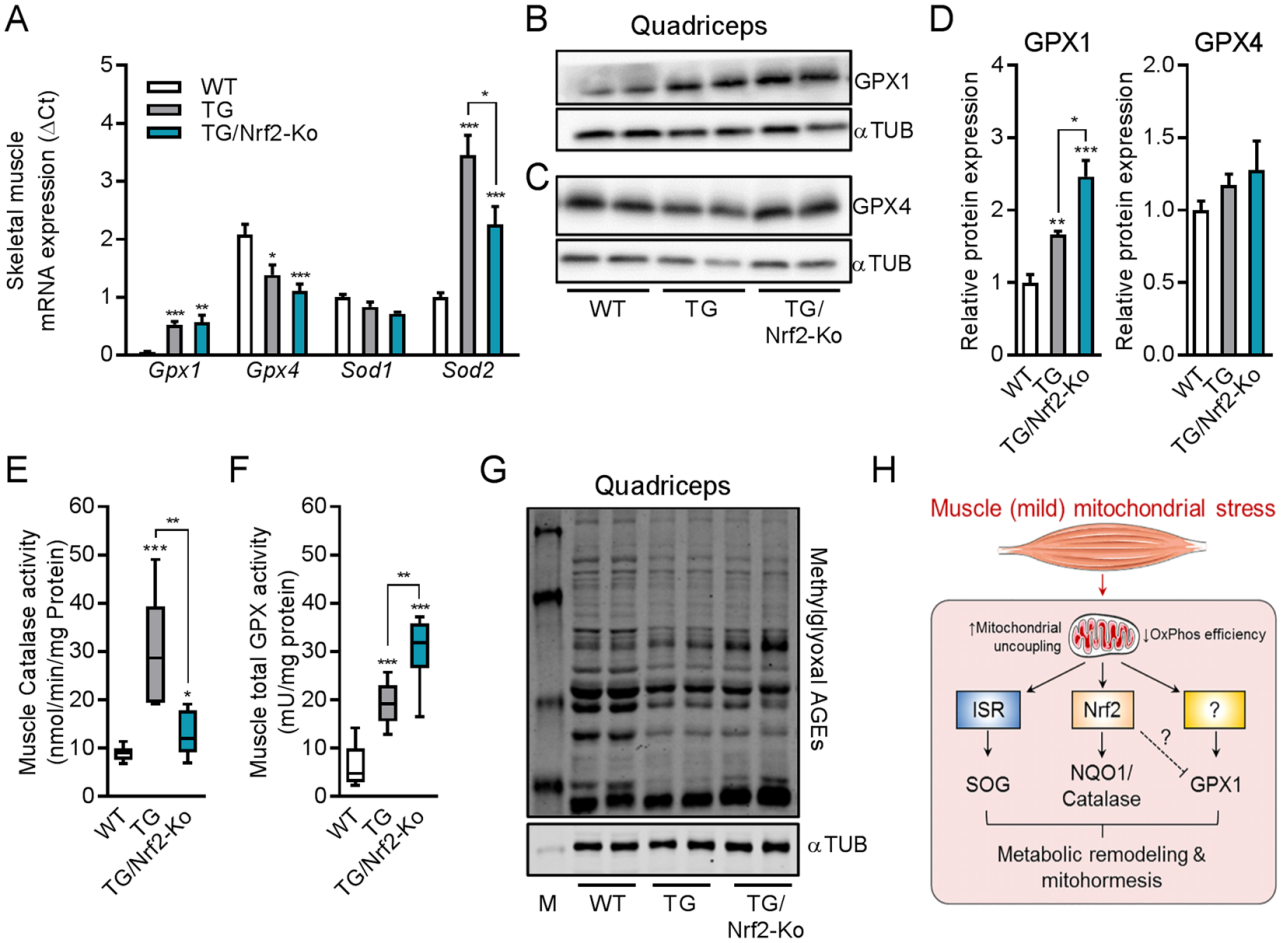
Induction of oxidative stress defense during muscle mild mitochondrial stress. (**A**) Skeletal muscle mRNA expression in quadriceps muscle of oxidative stress defense markers of 24 wks old animals (n = 8–10). (**B** and **C**) Representative immunoblots of glutathione peroxidase 1 (GPX1) and GPX4 from of cropped gels using αTubulin as loading control (2 samples shown out of 4–5 analyzed per group). (**D**) Quantification of GPX1 and GPX4 protein expression in quadriceps muscle from 24 wks old mice (4–5 per group). (**E**) Skeletal muscle catalase activity of 24 wks old animals (n = 5–10). (**F**) Skeletal muscle total GPX activity of 24 wks old animals (n = 7–10). (**G**) Representative immunoblots of methylglyoxal advanced glycation end-products (AGEs) in quadriceps muscle from 24 wks old mice using αTubulin (αTUB) as loading control (2 samples shown out of 4–5 analyzed per group). All absolute values are expressed as box-and-whisker plots, relative or normalized data are expressed as means + SEM; *p < 0.05; **p < 0.01; ***p < 0.001. (**H**) Graphical summary of Nrf2-dependent and Nrf2-independent adaptive cell-autonomous stress response pathways during respiratory uncoupling-induced muscle (mild) mitochondrial stress. This cartoon was created using Servier Medical Art (http://smart.servier.com).

Serine is an important precursor for the selenocysteine moiety of glutathione peroxidase^56^. As previous studies demonstrated the induction of the serine-glutathione biosynthetic pathway in response to OXPHOS inefficiency^32^ or mtDNA replication disorders^35^, we further analyzed key oxidative stress defense marker genes. We found that skeletal muscle mRNA expression of glutathione peroxidase 1 (*Gpx1)* and superoxide dismutase 2 (*Sod2*) was induced in muscle of TG mice which was preserved in TG/Nrf2-Ko mice (Fig. 5A). By contrast, mRNA expression of isoforms *Gpx4* and *Sod1* were reduced or not affected, respectively. Moreover, GPX1 protein expression was increased in skeletal muscle of TG mice and even higher in TG/Nrf2-Ko animals, while GPX4 protein was unchanged (Fig. 5B–D). Previously, we showed that muscle respiratory uncoupling promotes catalase^30^ and total GPX^32^ enzyme activity. Here, we confirmed elevated catalase activity in TG mice. This was abolished in TG/Nrf2-Ko animals (Fig. 5E). Interestingly, total muscle GPX activity, in accordance with gene and protein expression of GPX1, was even more strongly elevated in TG/Nrf2-Ko animals than in TG compared to WT (Fig. 5F). Finally, methylglyoxal related advanced glycation end products (AGEs), one endpoint of increased cellular oxidative stress, were not different in TG versus TG/Nrf2-Ko mice, indicating a maintained muscle redox balance (Fig. 5G). Thus, we here show that although Nrf2 mediates NQO1 and catalase activation, it is not required for the induction of key SOG pathway genes and even seems to partly suppress GPX1 activity during muscle metabolic remodeling and mitohormesis induced by mitochondrial uncoupling (Fig. 5H).

## Discussion

Nrf2 is a well-known determinant in the maintenance of redox homeostasis but so far, its role in muscle mitochondrial stress conditions had not been investigated. In the present study, we confirm and extend our previous data demonstrating that mitochondrial OXPHOS inefficiency caused by respiratory uncoupling induces several key antioxidant enzymes such as NQO1, catalase and GPX1 in skeletal muscle of TG animals. This is consistent with a mild uncoupling-induced delay of age-related disease^28,29,57^ and the concept of mitohormesis^58^. Notably, while muscle NQO1 and catalase induction are strongly Nrf2-dependent, we show that the loss of Nrf2 action does not prevent the increase of muscle GPX1 in TG mice. On the contrary, loss of Nrf2 even resulted in a further augmentation of GPX activity in response to muscle mitochondrial uncoupling. Furthermore, under conditions of mild mitochondrial perturbation, Nrf2 is not required to maintain skeletal muscle functional integrity and mitochondrial biogenesis despite a reduced mitochondrial respiratory capacity, probably due to the preserved AMPK activation. In addition, we demonstrate that ablation of Nrf2 activity in TG mice does not prevent the induction of key transcriptional and protein signatures of muscle UPR as well as ISR including key enzymes of the SOG pathway and the induction of stress induced cytokines.

Modulation of Nrf2 activity affects mitochondrial physiology, including respiration, ATP production, structural integrity and dynamics^59^. However, protein expression of mitochondrial respiratory complex subunits is not affected in muscle of unchallenged Nrf2-Ko mice^50^. In contrast, *in vitro* analyses using murine neurons and embryonic fibroblasts showed that Nrf2 deletion impairs mitochondrial respiration and activity of respiratory complexes^23^. Interestingly, this was related to a lower mitochondrial pool of NADH (complex I substrate) in Nrf2-Ko cells suggesting a less efficient production of NADH in the tricarboxylic acid (TCA) cycle. As part of metabolic adaptation, increased TCA cycle flux and basal mitochondrial uncoupling was observed in muscle of endurance trained individuals^60^. Moreover, a recent study reported that an impaired TCA cycle flux is a central mechanism of restricted OXPHOS capacity in McArdle disease, an autosomal recessive myopathy caused by mutations in the muscle isoform of glycogen phosphorylase^61^. Notably, Nrf2 was shown to regulate skeletal muscle glycogen metabolism^62^. We here show that the genetic ablation of Nrf2 in TG mice further reduces the mitochondrial OXPHOS capacity. However, whether Nrf2 is directly involved in the TCA cycle efficiency and OXPHOS adaptation in response to mild mitochondrial stress remains to be elucidated.

Recent studies uncovered a robust transcriptional and metabolic remodeling in patients^35^, mice^32,34^ and cell lines^55^ with mtDNA defects and mitochondrial dysfunction. Importantly, most of the up-regulated mitochondrial stress-induced genes carry a conserved amino acid response element (AARE) in their upstream regulatory region^34^, which is a binding site for ATF transcription factors. This cell-autonomous stress response involves components of amino acid metabolism (*Psat1*, PHGDH), one-carbon metabolism (*Mthfd2*), as well as mitochondrial stress-induced cytokines (FGF21, GDF15), recently referred to as the ´integrated mitochondrial stress response‘ (ISRmt)^63^. Notably, the crucial step in the signaling pathway of this adaptive ISRmt is the phosphorylation of eIF2α, which leads to a decrease in global protein synthesis and the induction of selected genes, including the transcription factor ATF4^64^. A recent study by DeNicola *et al*. demonstrated that the ATF4-mediated metabolic reprogramming and SOG pathway induction in cancer cells requires Nrf2 action^37^. However, while loss of Nrf2 further increased the expression of ER chaperone BIP and phosphorylation of eIF2α, we here provide evidence that the induction of muscle ISRmt, including markers of SOG pathway as well as the induction of FGF21 and GDF15 does not require Nrf2 action. Noteworthy, a recent study uncovered the mechanistic target of rapamycin complex 1 (mTORC1) as key regulator of muscle ATF4-mediated ISRmt and that treatment with rapamycin (mTORC1 inhibitor) reversed progression of mitochondrial myopathy in Deletor mice^65^. However, mild OXPHOS inefficiency was shown to activate AMPK in muscle of TG mice^29,42^. This activation of AMPK, which is still preserved in TG/Nrf2-ko mice, seems to be crucial for maintenance of muscle functional integrity since the suppression of AMPK in TG mice caused severe degenerative changes of skeletal muscle morphology and resulted in a greatly compromised exercise capacity^42^. AMPK is reported as potent negative regulator of mTORC1 activity^66^ which is in line with our previous observation that the mTOR pathway is not induced in respiratory uncoupled muscle^30^. Thereby, a potential mTORC1-independent regulation of skeletal muscle ISRmt should be addressed in future studies.

As we observed the highest GPX activity and GPX1 expression in respiratory uncoupled muscles without functional Nrf2. This might suggest that there is a need for compensating the loss of Nrf2 action in order to counterbalance mitochondrial dysfunction-induced oxidative stress. In turn, the presence of Nrf2 seems to put a break on the maximum activation of GPX activity. Further, it could be argued that GPX1 up-regulation could be an attempt to compensate for the loss of antioxidant Nrf2 target genes upon genetic ablation of Nrf2. The other way around, it is well-established that Nrf2 becomes activated when selenoprotein expression, including GPX1, is impaired^67^. This suggests a rather complex, indirect involvement of Nrf2 in skeletal muscle oxidative stress defense during mitochondrial dysfunction. Glutathione metabolism plays a critical role in skeletal muscle oxidative stress defense system^68^. We and others have already demonstrated an induction of glutathione biosynthesis and metabolism in response to OXPHOS inefficiency^31,32^ or mtDNA replication disorders^35^. Thus, our present results on muscle GPX1 strongly emphasize the importance of the glutathione system including GPX activity for muscle redox homeostasis and cell survival. GPX1 reduces hydrogen peroxide or small lipid peroxides using electrons provided by reduced glutathione (GSH)^69^. Interestingly, GPX1-deficient mice are healthy and fertile, suggesting a limited antioxidant role for GPX1 under normal physiological conditions^70^. However, in response to mitochondrial dysfunction cellular GSH levels are 1000-fold higher than other intracellular redox couples^71^. Moreover, GPX1 is important for adult muscle progenitor cell function and crucial for the integrity of muscle differentiation^72^. In 2006, the group of Bruce Spiegelman demonstrated that PGC1α is essential for the induction of ROS-detoxifying enzymes, including GPX1 and SOD2^73^. Consistent with this, previous studies could show an induction of muscle PGC1α expression in response to OXPHOS inefficiency^29,42^. Notably, mitochondrial complex IV (COX) deficiency is corrected by PGC1α activation^74^. Another recent *in vitro* study showed a translational regulation of GPX1 and GPX4 by the mTOR pathway^75^. However, as mentioned above, the mTOR pathway does not appear to be induced in respiratory uncoupled muscle^30^.

Generally, that raises the question whether the induction of endogenous antioxidant defense systems may help to delay disease progression or treat mitochondrial and age-related disorders? Previous studies showed that skeletal muscle-targeted mild respiratory uncoupling promotes survival and diminishes age-related diseases^28,29,57^. Overexpression of mitochondrial catalase in transgenic mice extended lifespan with reduced oxidative damage, mtDNA deletions, and delayed cardiac pathology^76^. Treatment of *mdx* mice, a mouse model for muscular dystrophy, with the Nrf2 activating compound sulforaphane showed a protection from oxidative damage and improved muscle performance due to the activation of NQO1^77^. Moreover, genetic ablation of Nrf2 enhances oxidative stress and impairs redox homeostasis in muscle of a dysferlin-deficient mouse model for early adult-onset, progressive muscular dystrophies^78^. It was further shown that NQO1 is crucial for maintenance of mitochondrial integrity in ageing^79^. An active lifestyle is able to conserve muscle redox status in elderly people by activation of Nrf2 signaling as shown by analyzing human muscle biopsies^80^. Future work is needed to investigate the molecular mechanisms of GPX1 induction during mitochondrial dysfunction and the role of Nrf2 in age-related disease.

In conclusion, our data emphasize the importance of a functional induction of oxidative stress defense in response to mitochondrial OXPHOS inefficiency. These observations open new perspectives on strategies increasing endogenous antioxidant defense mechanisms for therapy of mitochondrial and age-related metabolic disease. As key regulatory processes remain poorly understood, further insights e.g. from the comparison of different mouse models with mitochondrial dysfunction are required to improve our understanding of the metabolic remodeling and muscle mitohormesis that promote cellular recovery and survival.

## Material and Methods

### Animals and experimental setup

Animal experiments have been approved by the ethics committee of the Ministry of Agriculture and Environment (State Brandenburg, Germany) and all methods were carried out in accordance to permission number GZ V3-2437-18-2016. Adult male and female mice were used for the animal experiments that were group-housed and random-caged with *ad libitum* access to a standard chow-diet (Sniff, Soest, Germany) and water at 23 °C and a 12:12 h dark-light cycle. HSA-Ucp1-transgenic mice (TG)^26^ were crossed with Nrf2-knockout mice (Nrf2-Ko)^38^ kindly provided by Masayuki Yamamoto (Tohoku University Graduate School of Medicine) to generate the four experimental genotypes: wildtype (WT), Nrf2-Ko, human skeletal actin (HSA)-UCP1-transgenic (TG) and TG/Nrf2-Ko mice. For the experiments female and male animals were used and sacrificed in the end of the experiment after 2 h food withdrawal to collect plasma samples and organs. Unless stated differently, all data are from 24 wks old mice.

### *In vivo* phenotyping

For body composition measurement, quantitative magnetic resonance (QMR, EchoMRI 2012 Body Composition Analyzer, Houston, USA) was used. Voluntary running wheel activity was determined by IR motion detectors (TSE Systems GmbH, Homburg, Germany). Grip strength was measured using a Grip strength meter (BIOSEB).

### Plasma analysis

Random plasma insulin levels were measured by an ultra-sensitive ELISA assay (DRG Instruments GmbH, Germany) and plasma triglycerides, free fatty acids and total cholesterol were determined using an automated analyzer (Cobas Mira S, Hoffmann-La Roche, Basel, Switzerland) with the appropriate commercially available reagent kits (triglycerides, cholesterol CP, ABX, Montpellier, France; and NEFA; HR, Wako, Neuss, Germany). The plasma concentration of the circulating factors FGF21 and GDF15 were determined by sandwich ELISA (Mouse/Rat FGF-21 & Mouse/Rat GDF15 Quantikine ELISA Kit, R&D Systems) following manufacturer instructions.

### Enzyme activity analysis

Total glutathione peroxidase (GPX) in skeletal muscle was determined as described elsewhere using a GSH reductase-coupled test by photometric detection of µmol NADPH consumption per minute at 340 nm^81^. NAD(P)H:Chinon-Oxidoreductase 1 (NQO1) activity in muscle and liver were measured as described before^82^. In short, the NQO1 dependent production of menadiol is determined by spectrophotometricially detection of MTT-formazan that is formed by menadiol at 590 nm. Citrate synthase activity was determined spectrometrically as described previously by detection of DTNB formation at 412 nm^44^. Catalase activity was measured using the catalase assay kit (Cayman chemical, USA) and performed following the manufactures instructions.

### RNA isolation and gene expression analysis

Isolation of RNA and quantitative real-time PCR was performed as described previously^43^. For muscle gene expression analysis quadriceps muscle was used and calculated as dCT using hypoxanthine-guanine phosphoribosyltransferase (*Hprt)* for normalization. Primer sequences can be found in supplemental material and methods.

### Histology and Western blotting

Tissues were fixed in 4% formaldehyde and embedded in paraffin before 2 µm slices were cut for hematoxylin-eosin staining (Roth, Fluka). Protein isolation and immunoblotting from quadriceps muscle was performed as previously described^32^. If not stated differently, immunoblots were cropped from the same gel. The following antibodies were used for immunoblots: αTubulin (T6074, Sigma Aldrich), phospho-AMPK^Thr172^ (#2531, Cell Signaling), total AMPK (#2603, Cell Signaling), BIP (#3183, Cell Signaling), phospho-eIF2α^Ser51^ (#3597, Cell Signaling), total eIF2α (#5324, Cell Signaling), GPX1 (AF3798-SP, R&D Systems), GPX4 (ab125066, Abcam), IRE1α (#3294, Cell Signaling), Methylglyoxal (STA-011, Cell Biolabs), MFN2 (#9482, Cell Signaling), OXPHOS (#MS60, MitoSciences/Abcam), PGC1α (ab71130, Abcam), PHGDH (14719-1-AP, Proteintech).

### Skeletal muscle mitochondrial respiration

Mitochondrial respiration analysis was performed in soleus muscle as previously described using the high resolution Oxygraph-2k (OROBOROS Instruments, Innsbruck, Austria)^42^. Briefly, muscle samples were gently dissected, immediately placed in ice-cold biopsy preservation medium (BIOPS)^51^, separated with a pair of fine-tipped forceps, and finally permeabilized with saponin (50 µg/ml) for 30 min at 4 °C. After permeabilization, muscle fibers were washed in mitochondrial respiration medium (Mir05, 0,5 mM EGTA, 3 mM MgCl_2_•6H_2_O, 60 mM K-lactobionate, 20 mM Taurine, 10 mM KH_2_PO_4_, 20 mM HEPES, 110 mM Sucrose, 1 g/l fatty acid free BSA) for 10 min at 4 °C and kept on ice until analysis. Respiratory capacity was analyzed performing a multiple substrate-uncoupler-inhibitor titration (SUIT) protocol^52,53^ at 37 °C in a hyperoxygenated environment (see representative trace Fig. 3A) using following substrate concentrations: 2 mM Malate + 5 mM Pyruvate (LEAK respiration), 5 mM ADP (N-OXPHOS capacity), 10 µM cytochrome c (integrity of outer mt-membrane), 10 mM glutamate (N-OXPHOS capacity), 10 mM succinate (NS-OXPHOS capacity), 0.5 µM FCCP (NS-ETS capacity, ETS_CI&CII_), 0.5 µM rotenone (S-ETS capacity, ETS_CII_), 2.5 µM antimycin A (residual oxygen consumption, ROX). Oxygen flux levels were normalized to muscle wet weight of dry blotted fiber bundles.

### Statistical analysis

All absolute values are expressed as box-and-whisker plots, relative or normalized data are expressed as means + standard error of the mean (SEM). GraphPad Prism version 7 (Graphpad Software, San Diego, CA, USA) was used for statistical analysis, with Student’s t test being used to compare the two groups if normally distributed. One-way ANOVA (repeated measures, matched values) followed by the Bonferroni post hoc test was used for body weight, body composition analysis. *P* values < 0.05 were considered significantly different. Means with asterisk are significantly different to WT or indicated group.

### Data availability

The datasets generated during and/or analysed during the current study are available from the corresponding author on reasonable request.

## Electronic supplementary material

Supplementary information
