# Author Correction: Partial involvement of Nrf2 in skeletal muscle mitohormesis as an adaptive response to mitochondrial uncoupling

**DOI:** 10.1038/s41598-018-29610-4

**Published:** 2018-07-24

**Authors:** Verena Coleman, Piangkwan Sa-Nguanmoo, Jeannette Koenig, Tim J. Schulz, Tilman Grune, Susanne Klaus, Anna P. Kipp, Mario Ost

**Affiliations:** 1 Department of Physiology of Energy Metabolism, German Institute of Human Nutrition Potsdam-Rehbrücke, 14558 Nuthetal, Germany; 2 Department of Molecular Toxicology, German Institute of Human Nutrition Potsdam-Rehbrücke, 14558 Nuthetal, Germany; 3 Department of Adipocyte Development and Nutrition, German Institute of Human Nutrition Potsdam-Rehbrücke, 14558 Nuthetal, Germany; 4 Department of Physiology, Chang Mai University, Chang Mai, Thailand; 5 Department Molecular Nutritional Physiology, Friedrich Schiller University Jena, Jena, 07743 Germany

Correction to: *Scientific Reports* 10.1038/s41598-018-20901-4, published online 05 February 2018

The Acknowledgements section in this Article is incomplete.

“The authors would like to thank Antje Sylvester, Petra Albrecht, Carola Gehrmann and Stefanie Deubel for their excellent technical assistances.”

should read:

“The authors would like to thank Antje Sylvester, Petra Albrecht, Carola Gehrmann and Stefanie Deubel for their excellent technical assistances. The publication of this article was funded by the Open Access Fund of the Leibniz Association.”

