# Supplementary material for: Partial involvement of Nrf2 in skeletal muscle mitohormesis as an adaptive response to mitochondrial uncoupling
Source: Sci Rep. 2018 Feb 5;8:2446. doi: 10.1038/s41598-018-20901-4 (PMC5799251; doi:10.1038/s41598-018-20901-4)
Supplement: Supplementary file 1 — Supplementary information [file 41598_2018_20901_MOESM1_ESM.pdf]

Supplementary information

Partial involvement of Nrf2 in skeletal muscle mitohormesis as an adaptive response to mitochondrial uncoupling

Verena Coleman[1], Piangkwan Sa-Nguanmoo[1,4], Jeannette Koenig[2], Tim J. Schulz[3], Tilman Grune[2], Susanne Klaus[1], Anna P. Kipp[5], Mario Ost[1]*

[1]Department of Physiology of Energy Metabolism, German Institute of Human Nutrition Potsdam-Rehbrücke, 14558 Nuthetal, Germany

[2]Department of Molecular Toxicology, German Institute of Human Nutrition Potsdam-Rehbrücke, 14558 Nuthetal, Germany

[3]Department of Adipocyte Development and Nutrition, German Institute of Human Nutrition Potsdam-Rehbrücke, 14558 Nuthetal, Germany

[4]Department of Physiology, Chang Mai University, Chang Mai, Thailand

[5]Department Molecular Nutritional Physiology, Friedrich Schiller University Jena, Germany

*Corresponding author: Mario Ost

E-mail: Mario.ost@dife.de

Mario Ost, PhD

Department of Physiology of Energy Metabolism

German Institute of Human Nutrition (DIfE), Potsdam-Rehbrücke

Arthur-Scheunert-Allee 114-116

14558 Nuthetal (Germany)

Tel: +49 33200 88-2430

E-mail: Mario.ost@dife.de

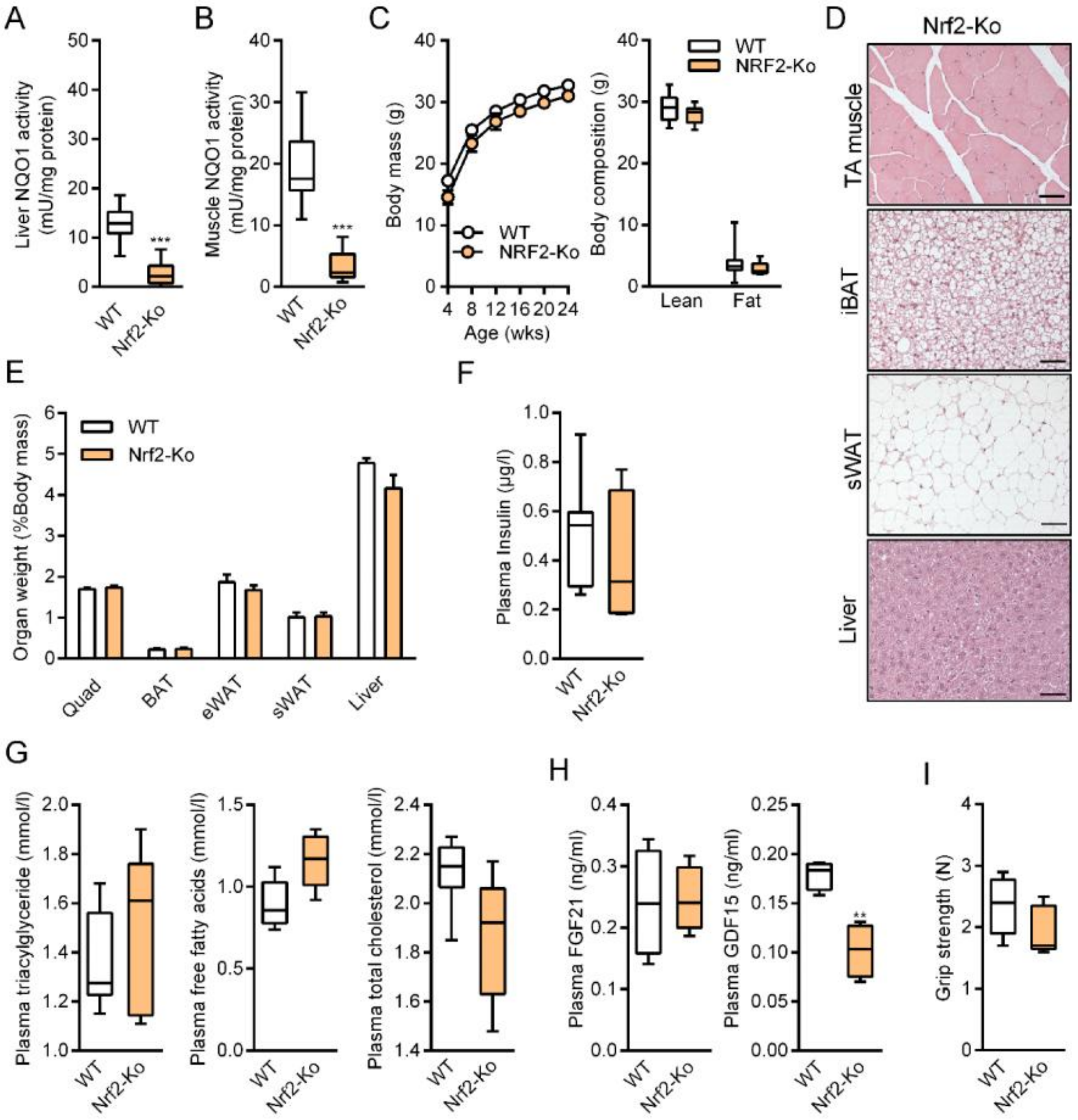

Figure S1. Phenotypic characterization of male Nrf2-Ko animals. (A) Body mass development from wks 4 to wks 24 and final body composition of 24 wks old animals on standard diet (n=5-10), (B) Liver and (C) Quadriceps muscle NQO1 activity (n=5-10) (D) relative quadriceps (Quad), interscapular brown adipose tissue (iBAT), epidydimal (eWAT) and subcutaneous white adipose tissue (sWAT) and liver weights and (E) H&E histological staining of Tibiales anterior (TA) muscle, iBAT, sWAT and liver of 24 wks old animals, bars represent 50 µm, (F) random fed plasma insulin levels, (G) plasma triacylglycerides, free fatty acids and total cholesterol, (H) plasma FGF21 and GDF15 levels of 24 wks old animals (n=5-10). All absolute values are expressed as box-and-whisker plots, relative or normalized data are expressed as means + SEM; $^{**}p<0.01$; $^{***}p<0.001$.

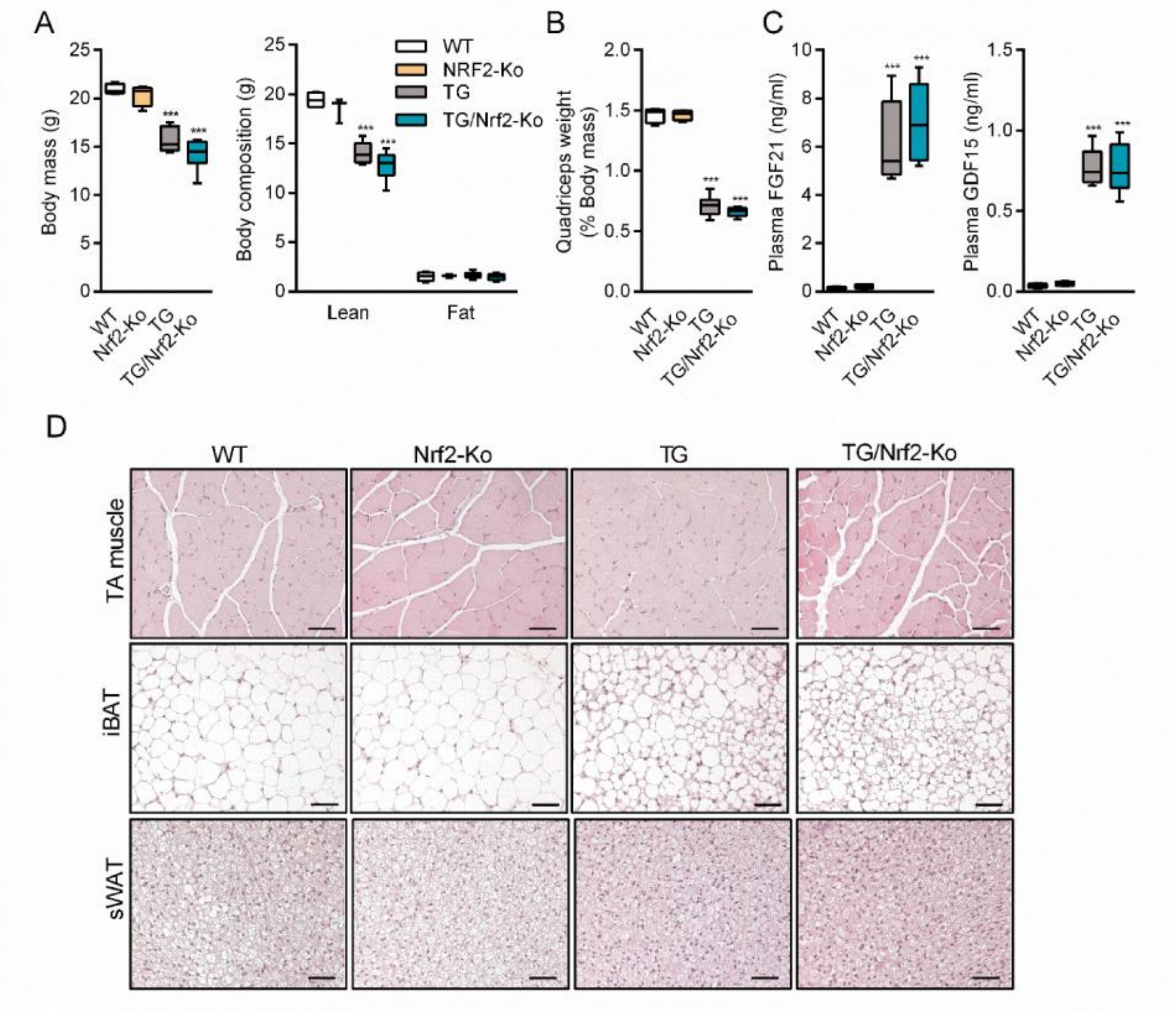

Figure S2. Phenotypic characterization of female TG/Nrf2-Ko animals. (A) Body mass and body composition, (B) relative quadriceps (Quad) weights, (C) plasma FGF21 and GDF15 levels, (D) H&E histological staining of Tibiales anterior (TA) muscle, interscapular brown adipose tissue (iBAT) and sWAT of 12-14 wks old female animals (n=3-7), bars represent 50 µm. All absolute values are expressed as box-and-whisker plots, relative or normalized data are expressed as means + SEM; ****p*<0.001.
